# China environmental economics: A political authority approach and neo-marxist perspective in the emission trading system (ETS) implementation

**DOI:** 10.1016/j.heliyon.2024.e40633

**Published:** 2024-11-22

**Authors:** Roseno Aji Affandi, Adrian

**Affiliations:** Bina Nusantara (Binus) University, Indonesia

**Keywords:** Environmental economics, China emission trading system, China economics, Neo-marxism, Political authority

## Abstract

The study focuses on the implementation of the Emissions Trading System (ETS) in China and its impact on the economy, using an environmental economic theory framework reinforced by the theory of political authority and neo-Marxist perspectives. The research find that The ETS policy has a long-term positive impact on the economy and the amount of carbon intensity. The Chinese government influence is very powerful and can ensure that all entities follow the rules of emissions trading, which facilitates policy enforcement. Emissions trading also provides an opportunity for the business world to invest and generate new ideas, which will produce better products in the future. The research argue that China is striving to align itself with global governance in order to secure its economic interests, employing a political authority approach as a component of the Marxist perspective. The study examines the decision-making process in choosing between the short-term and long-term impact on the Chinese economy following the implementation of the Emissions Trading System (ETS). The study uses qualitative research methodology, using secondary data from previous research.

## 1 Introduction

China is formulating a development strategy by including sustainable green economy development programs into its national agenda. China implemented its 12th Five-Year Economic Development Plan (2011–2015) strategy starting in 2011. China's 12th development strategy prioritizes the achievement of a green economy, which is measured by factors such as heightened investments in key green sectors, particularly in renewable technologies and energy. The government's policies and legislation support and strengthen these efforts. The Act aims to decrease carbon dioxide emissions primarily by implementing the Emissions Trading System (ETS) policy to promote emission reduction. China's goal to achieve carbon neutrality by 2060 and peaking carbon emissions by 2030 also includes regulations for the Emissions Trading System (ETS) [1].China initiated its significant action by trialing the ETS policy in seven provinces in 2013. The outcomes of this trial, which were considered satisfactory, led to its nationwide implementation by 2021 [2].

This study contends that the adoption of an Emissions Trading System (ETS) in China leads to a significant reduction in carbon emissions. This is evidenced by the declining carbon intensity observed from 2013 to 2018 [1]. The efficacy of ETS deployment is assessed by comparing regions that have not adopted it; these regions exhibit poorer outcomes than those utilizing ETS. These indications demonstrate that the ETS prototype can impact and improve the dynamics of carbon intensity.

Additionally, China is penalizing companies that violate pollution limits. The offender was fined one to three times the market price. The gap between ETS implementation and China's GDP is examined in this study. This application raises production costs, which affects sales prices and national income. This is important for China's economy, as exports are its primary source [3]. The cost of carbon emissions is a crucial factor in the execution of the ETS [2]. National economic growth is also affected by carbon emission prices. Wealthy countries and cities boost social output, consumption, and CO2 emissions [4]. Emissions Trading Systems (ETS) and corporate-government relations may harm production costs and national revenue. Note that ETS does not negatively affect GDP over time. It is important to understand how a communist-led authoritarian government is adopting global CO2 emission rules, which could raise manufacturing prices and reduce China's export advantage. This research is crucial to understanding global value-based economic development under China's communist regime.

Countries that experience significant per capita growth or substantial economic growth have a beneficial effect on enhancing human well-being and promoting sustainable development [5]. Nevertheless, the swift expansion engenders disparities among the industrial sector, state-owned firms, and private enterprises. Although it may sound unfavourable, proponents of market libertarianism contend that this approach yields superior outcomes in the long term [4].

The influence of government policies and laws on economic growth and environmental quality is significant. Emission standards, land use planning, and conservation measures are examples of environmental rules that can effectively mitigate the environmental harm caused by economic activity. In certain instances, a reciprocal relationship may exist between economic growth and environmental quality. For instance, policies that give priority to economic growth may result in immediate financial gains but ultimately lead to long-term environmental harm. Conversely, policies that place emphasis on safeguarding the environment may impose a temporary strain on economic growth, yet yield enduring advantages in the long run.

The correlation between economic growth and environmental quality, economic expansion has the ability to stimulate technological innovation, which in turn can enhance environmental quality. Advancements in renewable energy technologies, waste management, and pollution control have the potential to mitigate the adverse environmental consequences associated with economic activity. Within the framework of market dynamics, the consciousness of environmental concerns among customers has the potential to stimulate the demand for products and behaviors that are environmentally sustainable. Industries that prioritize sustainability and conservation can benefit from the creation of economic prospects.

The favourable correlation is substantiated by China's capacity to enact measures centered around the environment. Liberal and communist-based regimes exhibit distinct approaches in the development and execution of policies. Ideological disparities and variations in political structures contribute to divergent approaches to decision-making. For instance, governments in communist nations exert significant influence in the development and execution of policies. Governments frequently exercise influence over significant areas of the economy, such as banking, energy, and telecommunications, through the establishment of state-owned businesses. Furthermore, communist states prioritize state authority over the economy, centralized strategizing, and endeavors towards common objectives [6]. Policies frequently aim to attain social equality and promote the welfare of the proletariat. In communist governments, political decision-making tends to exhibit centralization, when authority is predominantly concentrated within the ruling party or the single party system. The process of decision-making may encompass hierarchical directives issued by party leaders or central planning entities. The execution of policies is frequently conducted via a hierarchical bureaucracy, where failure to comply with official directives can lead to penalties or punishments [7].

Policymaking in liberal states is typically characterized by decentralization and pluralism. The decision-making process encompasses a multitude of stakeholders, comprising elected officials, governmental entities, civil society organizations, and interest groups. Policy is typically informed by fundamental ideals, such the promotion of individual autonomy, the facilitation of free markets, and the imposition of little government interference in economic and social matters [6]. Government agencies are responsible for the enforcement of laws and regulations, while people and organizations have the ability to seek compensation through legal channels in the event that they perceive a violation of their rights [8].

When examining these disparities, it becomes evident that China is effectively executing the ETS program. Government controls play a crucial role in facilitating decision-making and coordinating the implementation of policies. The implementation of policies has the potential to expedite the attainment of emissions reduction and economic growth objectives. Furthermore, there exist environmental institutions at both the national and municipal levels that are responsible for supervising the execution of environmental laws, enforcing regulatory measures, and monitoring the extent of pollution. This entity enables the Chinese government to implement regulatory measures at both regional and national scales.

The study employs environmental economic theory to elucidate the correlation between global governance in environmental conservation and economies that operate harmoniously rather than antagonistically, aligning with the notion of a green economy. This study also uses the theory of political authority, substantiated by neo-Marxism to elucidate China's global governance. State-owned enterprises (SOE) are a component of China's state-centric strategy for implementing the ETS. The significance of state-owned enterprises in stimulating Chinese economic growth is evident through their dominance over critical sectors, ex including energy, finance, and transportation. This control enables the Chinese government influence on economic development and ensure stability in the management of important sectors (Yang et al., 2021).

This study posits that China possesses robust political authority, enabling it to effectively implement the ETS program. Moreover, with the implementation of a centralized policy, China may effectively execute a beneficial strategy of adaptation to the European Union Emissions Trading System (EU ETS). The perspective of Neo-Marxists posits that the process of decentralization will confer agency upon local communities and workers. The objective of this decentralization is to equitably distribute decision-making authority and diminish the consolidation of power among a select few elites. These techniques and perspectives align with the principles of environmental economic theory, which posits that economic activity has an influence on the environment. However, it is important to ascertain whether there exists a mutually beneficial relationship between these two factors. This study aims to assess the influence of the ETS implementation on China's economic growth and its correlation with the efficacy of carbon emission reduction. This study examines the implementation of environmental-based policies in communist countries, specifically focusing on China, which have been copied from liberal governments.

The use of environmental economics is known to affect on economic decline. However, this study examines economic and development policy methods that can combine economic growth with environmental considerations. This study is founded on Nick Hanley's book titled "Environmental Economics in Theory and Practice." The book elucidates that environmental economics theory establishes a connection between the influence of economic activities on the environment and examines the strategies and policy frameworks that can promote sustainable development. Environmental economics posits that natural resources are the primary means of facilitating human production and consumption (Hanley et al., 1997). Therefore, assessing the value of natural resources is a crucial factor, and enhancing the condition of the environment is imperative. Environmental economist contend that several methodologies can effectively tackle environmental issues [9]. There are many ways for a country to switch to sustainable energy. Governments can regulate natural resource use, such as carbon emissions. Another possibility is that fiscal like levying carbon taxes or giving tax credits to corporations that use emissions from state-owned industries or private companies. State fiscal instruments are used to intervene in markets to reduce carbon emissions.

The main objective of this study is to analyze the impact of the implementation of the Emission Trading System on economic growth in China and the effectiveness in reducing carbon emissions. This study looks at the implementation aspects of ETS from business institutions and time periods. Through this aspect, research analysis provides answers to research questions that have already been made. The research questions in this paper are as follows: "How does the ETS affect China's economic growth and sustainable development, taking environmental economic theory, political authority theory, and neo-Marxism perspective?". This research is crucial for examining the relationship between implementation and economic growth. Applying the notion of a liberal state within a communist state that embraces a green economy exhibits notable disparities. Ultimately, applying the idea in both instances of the nation creates discrepancies and contradictions that warrant careful ideological divisions, encompassing liberal and communist perspectives. The study employs environmental economics, political authority, and neo-Marxism perspective to examine the relationship between enhanced environmental quality and heightened economic growth in China.

## 2 Theory

The study on ETS in China incorporates two theories, specifically environmental, economic, and political authority. Political authority in global governance refers to the regulations and principles that establish the obligations and limitations of a specific political entity or agency. Political authority necessitates the existence of an institution that oversees decision-making for the collective benefit, hence defining political authority as the ability of a political entity to enforce its own regulations [10]. Political authority can be categorized into two distinct types: de facto and de jure [11]. De facto refers to a situation where the government possesses the actual ability to enforce compliance and citizens willingly adhere to it. The research utilized the notion of political authority to elucidate the implementation of ETS on a regional or national level in China.

The state-centric approach in current political and social science is rooted in neo-Marxism, which posits that all power is concentrated within the central government. Neo-Marxism posits that the primary agents of action are the governors, rulers, and political administrators[12]. When considering power, an essential element in influencing social and political behavior is the arrangement of state institutions and the policies that are passed down from previous generations. The greater the strength and capability of an administrative state, the higher the potential for achieving various objectives. The welfare state is anticipated to reflect the inherited structure of the nation. There are two main contributions to this method, specifically the emphasis on the influence of the interstate system on the ongoing implementation of public policy [13]. The second factor is the presence of a historiographical methodology that examines the process and circumstances involved in the formulation and execution of a policy. This approach elucidates the political legitimacy of China, a communist state, in implementing the ETS.

The study employs the environmental economic theory, alongside the theories of political authority and neo-Marxism, to elucidate the influence of an ETS policy on China's economic growth. This theory examines the impact of economic policies on the environment and the reciprocal influence of the environment on economic decision-making [14]. Regardless, economic activity will inevitably exert an influence on the environment. Due to the significant magnitude of this impact, it is necessary to conduct a study of the ETS legislation using environmental economic theory. In his book "Environmental Economics in Theory and Practice," Nick Hanley argues that policy initiatives should consider the potential for sustainable development and recognize the favourable consequences of such policies. If the aforementioned approach fails to result in a mutually beneficial outcome, then it can be concluded that the policy is detrimental to the environment. The theoretical framework is illustrated and explained as follows.

**Figure:**
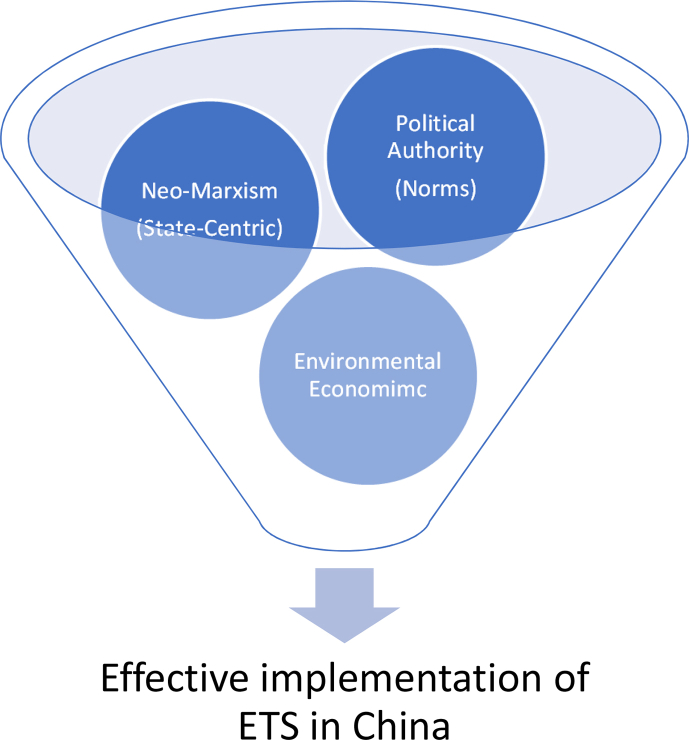

China, as a communist nation, has been enacting a range of environmental-oriented policies over an extended period of time. In recent decades, China's leadership has acknowledged the significance of environmental protection in light of the substantial environmental issues confronting the nation, despite the traditional emphasis of communism on economic equality and public ownership of resources. In certain instances and within specific nations, there exists a theoretical continuity between epistemic authority and political authority. This study primarily focuses on political authorities, aligning with the perspective of neo-Marxism. As a communist state, China employs epistemic authority exclusively to embrace a novel growth or economic trajectory, specifically, the implementation of a green economy. Epistemic authority pertains to the recognition or trustworthiness bestowed to a nation, individual, or entity, predicated upon their perceived knowledge, experience, or dependability within a specific field or realm of inquiry. Essentially, it refers to the acknowledgment that a State, individual, or source possesses the necessary knowledge and is thus regarded as a trustworthy source or authority on matters within their jurisdictional boundaries. This environmentally conscious economic movement originates from Western/liberal nations. China must adjust to this matter as their primary market is located in a liberal nation. The report posits that the implementation in question is also aligned with China's strategic objectives of fostering economic growth.

Currently, China holds the distinction of being the nation with the highest magnitude of commodity trade globally. By the year 2023, the aggregate worth of China's exports and imports had attained a sum of $5.9 trillion (Statista Research Department, 2024). China's status as the largest importer, along with its extensive network of partners, has resulted in a trade surplus on the export front. The trade balance plays a crucial role in determining the economic progress of a nation. China has generated substantial profits from the exportation of diverse items, particularly when it is rooted in an environmental economy.

Within the framework of policy implementation, the ETS is executed through the utilization of the Political Authority approach. Given that industrial actors in China primarily consist of state-owned enterprises (SOEs) and multinational corporations (MNCs), as well as big industrial industries resulting from foreign investment, we contend that China employs two complementary approaches simultaneously in response to global governance. The field of epistemics with regards to conformity with Foreign and Political Authority mostly centers on the nation itself.

The implementation of China's ETS policy is contingent upon the interplay of Political Authority, Neo-Marxism, and Environmental Economics. Being a communist state with a Chinese base, it possesses a formidable ability to implement policies by coercion. The primary aim is to ensure that the actors and commercial institutions partake in the policies set forth by the central government. Moreover, from the standpoint of neo-Marxism, China, as a nation that adopts a decentralized structure, possesses the ability to exert complete authority over its policies. The implementation of various top-down government programs by the Chinese government is being undertaken with the objective of addressing environmental issues. These programs frequently entail establishing ambitious objectives to mitigate pollution, enhance the utilization of renewable energy sources, and enhance the quality of air and water. China's policy implementation is facilitated by its country-centric strategy, which allows for swift and resolute actions. Authorities possess the ability to formulate policies without encountering substantial resistance from other political entities, hence expediting the execution of endeavors such as poverty alleviation efforts, environmental regulations, and industrial policies. China's centralized control enables it to efficiently address urgent issues and achieve its development objectives within a relatively brief timeframe.

By using political authority and implementing neo-marxist principles, we may achieve the objective of the ETS to decrease carbon emissions while simultaneously promoting China's economic growth. The concept under consideration holds significant importance within the realm of environmental economic theory, as it posits that an economic policy pertaining to the environment can be deemed favourable when it engenders a positive correlation. The comprehension of the implementation of the ETS in China, a communist state, necessitates the consideration of three significant theories and methodologies.

## 3 Literature review

This study expands upon a prior investigation published in the Journal titled "Can the pilot emission trading system effectively align emission reduction and economic development objectives in China?". The study examines the effects of implementing an Emissions Trading System (ETS) policy on the economic growth of China and the disparities between private enterprises and state-owned enterprises in executing the Emission Trading System (ETS) and its repercussions on investment. Our research employs a developmental political theory approach to analyze the adoption of global governance in environmental challenges by communist governments, particularly those influenced by Western or liberal states.

Regarding the correlation between the economy and the environment, there is considerable controversy over the potential for a mutually beneficial influence on both domains. Experts are studying to assess the economic implications of implementing ETS in China. China has been launching ETS prototypes since 2013 in terms of practical experience [15].Nevertheless, the experimental deployment of the ETS in the current year was limited to only seven regions, namely Tianjin, Chongqing, Beijing, Guangdong, Hubei, Shanghai, and Shenzhen. Undoubtedly, China, being the primary entity in the International ETS, must be ready to confront a significant threat if and when it arises. Hao Ran Zhang's research revealed a substantial reduction in carbon emissions within the ETS trial area located in Chongqing, Hubei, and Tianjin. The drop in intensity is a result of the efficacy of policy implementation. In addition, these accomplishments also contribute to a certain degree of advancement in economic growth. This policy promotes the advancement of technical innovation by improving the pathway for future development [15].

The experts claim that the adoption of ETS effectively mitigates carbon emissions and fosters cooperative air governance. Hao Ran Zhang analyzed various focal points of the ETS pilot and found that the magnitude of carbon emissions is diminishing. The installation of this prototype project has effectively eradicated carbon emissions [15].In terms of the economy, the ETS has a positive effect on China's GDP. Guangdong and Shanghai exhibit greater values of root mean square percent error (RMSPE) compared to Tianjin, Hubei, Chongqing, and Beijing. By implementing the prototype ETS, there is a definitive disparity of 74.5 billion yuan between Beijing's average annual "real" and "synthetic" GDP, with an average fluctuation of 2.82 %. The disparity is significant enough to be observable compared to the previous average of 5.9 billion yuan every year, with subsequent swings averaging 0.87 % [15].

Similarly, the introduction of the ETS pilot project has expedited the economic growth of Tianjin, Chongqing, and Hubei. Among these three regions, Hubei has the biggest absolute average annual difference, which was recorded at 269 billion yuan from 2014 to 2018. After the policy was implemented, Chongqing saw a substantial average variation of 11.63 %. The project partially achieved economic prosperity in an indirect manner [15].

Within the framework of China's economy, the significance of firms is paramount in bolstering economic expansion. Investments should be executed with efficiency, ensuring that there is no superfluous allocation of resources. The adoption of ETS has a beneficial impact on the effectiveness of the company's investments and can impede excessive investment [2]. The investment efficiency has experienced a substantial growth, and the degree of governance in state-owned businesses surpasses that of privately-owned companies. However, the implementation of policies in China has a significant and ever-changing effect on investment spending. During challenging circumstances imposed by the government, there is a temporary rise in the expenses associated with green innovation initiatives. This action demonstrates the need for modifications to industry green technology developments in response to environmental policy [2].

ETS analysis improves Chinese investment. ETS implementation has four effects: In the beginning, business innovation and green patents rise. Additionally, corporate emissions have fallen and spurred ecologically friendly technical advances. Corporations could offer carbon allowances to boost revenue and investment efficiency. Environmental constraints have a compensating effect that increases investment efficiency through innovation. This strategy may also encourage companies to optimize input and technology use, improving investment efficiency. ETS strengthens the company's external governance. ETS companies can give more transaction details, which helps them improve investment efficiency [2].The adoption of the Emissions Trading System (ETS) effectively achieved a reduction in carbon emissions. The reduction achieved is 24.2 %. The Chinese government must prioritize the establishment of intermediaries, particularly inside commercial banks and carbon asset management agencies, in order to facilitate its development goals [16].

State-owned firms and the private sector are crucial to balancing economic growth and environmental sustainability in the ETS's successful implementation. The authoritarian Chinese government has forced these two commercial groups to follow the rules when exercising their rights and completing their commitments. China's environmental law enforcement differs due to entities' goals and capacities. Environmental governance system political jurisdiction over local and national governments differs. China must share authority and obligations to build its economy due to its geographical diversity. China's state-centric philosophy allows policy implementation to be distributed among areas, taking into account their differences [17].

The province's priority is to optimize the political performance of administrations. The presence of regional diversity results in a range of pressures that frequently fall behind the central government's overarching objectives in terms of environmental conservation and economic progress. As an illustration, the central government aims to prioritize increasing the national income and fostering economic growth. Consequently, local governments may be compelled to cease their efforts in safeguarding the environment to prioritize economic expansion. Conversely, local governments may opt to prioritize environmental preservation over economic growth. Both of these possibilities are in direct opposition to the central government's objective of achieving a harmonious equilibrium between economic expansion and environmental conservation. Put simply, local governments may engage in activities that may not entirely adhere to national environmental standards or may prioritize a single objective.

The article written by Lili Jiang also discusses the significant positive correlation between the development of carbon finance and high-quality economic growth in China. Provinces with stronger carbon finance systems show better economic performance in many aspects, including coordinated, environmentally friendly, and shared development. The positive impact of carbon finance is more noticeable in economically prosperous eastern provinces such as Beijing and Shanghai compared to less developed regions. The authors argue that carbon financing mechanisms such as emissions trading schemes and eco-friendly bonds provide vital capital to support environmentally friendly technologies, improved energy efficiency, and sustainable practices in industry. It promotes the transition to a low-carbon economy, improves environmental well-being and resource [18]Moreover, the carbon trading scheme actually drives a transition towards more environmentally friendly energy sources in the participating regions. This means less dependence on fossil fuels like coal and increased use of more environmentally friendly alternative fuels such as renewable energy [19].

From the entire review of the literature, we can find strong arguments to explain that the ETS is a powerful tool to boost the economy and create new technological innovation. In the end, the intensity of carbon emissions will decrease by adhering to the policy.

## 4 Material and methods

In order to address the research inquiries, the investigation follows procedures: (1) Empirical Economic Environmental: ETS Political Authority Approach. This section focuses on China's development strategy, specifically on the political authority approach in China and Romania in implementation of the ETS. (2) The China Environmental Economic Development Challenges. This section focuses on the obstacles China faces in achieving its immediate and long-term economic goals and the efforts made by business institutions to create an Emissions Trading System (ETS). (3) Analyzing China’s ETS Implementation with A Political Authority Approach and Neo-Marxist Perspective. This section examines China's adoption of an Emissions Trading System (ETS) using a state-centric approach that relies on strong political authority to promote environmental economics. The research methodology employed is qualitative, utilizing secondary data obtained from prior studies on.

1. The Article titled "Carbon emissions trading system and investment efficiency: Evidence from China" discovered that the findings of the ETS policy harmed investment and contended that the implementation of ETS reduced the efficacy of both state-owned enterprises and private investment. Actively executing an ETS helps a company maximize investment efficiency. A company is profitable and invests efficiently when it benefits both itself and the nation. China faces challenges in adopting this ETS, but the results are positive. China is less seasoned than the EU despite its expertise. Thus, state-owned firms and private corporations are essential to China's sustained development.
2. "Can the pilot emission trading system coordinate the relationship between emission reduction and economic development goals in China?" The research reveals that various Chinese regions that adopted the ETS pilot program achieved a reduction in carbon emissions and a moderate increase in economic growth. It also suggests that the ETS pilot program has the potential to enhance energy efficiency. The region experiences annual fluctuations on average. The data demonstrates that the implementation of the ETS remains effective, as seen by a concurrent decrease in carbon intensity. The successful reduction of pollutants serves as an exemplary model for other companies to follow in their pursuit of sustainable development.
3. Title of the article: "Centralization or decentralization? the impact of different distributions of authority on China’s environmental regulation": The study examines the effects of varying distributions of authority on China's environmental regulation, specifically focusing on the differences in the implementation of environmental policies across different regions." The focal point in this publication is the disparity between the functions of the central and local government.

## 5 Result

The literature review used in this study has a correlation in the interpretation of the impact of positive ETS policies. Nevertheless, some literature does not clearly sufficiently discuss the actions of states sufficiently tough in implementing ETS policy. Political authority theory and neo-Marxism see that China has the power to force actors and institutions to abide by the ETS policy. With this coercive and centralized policy, the achievement of China's goals will quickly come true. On the other hand, the current China shows an improvement in the production efficiency of the company, both in the short and long term. Produced products become more competitive and thus contribute to improving the rate of the Chinese economy [20]. The research implications and contributions towards China's political economy theory are specifically focused and limited to the study of emission issues. Not only democratic countries, but also communist countries, such as China, can effectively implement life environmental policies in coercive ways in the interests of life environment. Entrepreneurs must abide by Chinese rules and policies according to the desired system. China's environmentally-conscious business and innovation strategy has experienced significant growth, reaching 90 %. This is due to the high consumption awareness of the environment. China will experience significant economic growth as a result of its vast market creation.

Companies with strong ESG practices can demonstrate their commitment to responsible business practices and long-term sustainability. These positive signals can boost the company's reputation, attract responsible investors, and potentially lower capital costs [21]. So, it's important to keep considering the importance of the whole pivot for long-term success. It also potentially leads to improved performance during crises when relations with stakeholders. Companies involved in the ETS policy also showed an improvement in the various environmental, social, and governance dimensions compared to non-participating companies (S. [22]). Other benefits are encouraging companies to explore and adopt environmentally friendly technologies, which lead to reduced emissions and potential cost savings through improved resource efficiency.

The research find that The Chinese nation benefits from environmental economic interests as they attract investment and foster technological innovation, resulting in a favourable outcome. The drop in carbon emissions in China has led to the creation of a mutually beneficial solution. Furthermore, China's strong emphasis on state-centric political authority greatly reinforces the implementation of the ETS. China's burgeoning export activity exerts a significant influence on its economic growth. This expansion encompasses augmented Gross National Product (GNP), amplified production from both private enterprises and state-owned enterprises, and the generation of employment opportunities. In addition, China's export quality and competitiveness are enhanced by the transfer of technology and expertise facilitated by international commerce.

## 6 Discussion

### 6.1 Empirical Economic Environmental: ETS political authority approach

This section provides an empirical application of the Emission Trading System (ETS) model in the European Union, specifically focusing on a case study conducted in Romania. Subsequently, this part examines the implementation of ETS in Romania and China, both of which have a same political system: communism, which is only single party to rule the state. The EU has implemented a Kyoto Protocol-based carbon trading scheme. Environmental restrictions and directives in Europe are strict by implementing incentive-based tax policy. EU policies have affected the whole commercial sector under its jurisdiction. The EU's ETS uses 'cap and trade' to control emission allowances. Companies can emit one metric ton of CO2eq per benefit. To compensate for pollution, companies must pay. If benefit quantity is unsuitable, a heavy penalty will be enforced. The company can buy the allowance from the EU carbon market. However, the corporation may receive a specified allowance for free. Companies can market their needs-based benefits. The company may trade future emissions. In order to regulate emissions from energy-intensive firms, commercial enterprises in Europe, and power plants, the EU ETS was created. The study examines how an ETS policy affects China's economic growth using environmental economic, political authority theory, and neo-Marxism perspective. This theory investigates how economic policies affect the environment and when the environment affects economic decisions. Economic activity affects the environment regardless. It's vital to evaluate ETS legislation using environmental economic theory due to its large influence. In his book "Environmental Economics in Theory and Practice," Nick Hanley argues that policy initiatives should consider the potential for sustainable development and recognize the favourable consequences of such policies. If the aforementioned course of action fails to result in a mutually beneficial outcome, then it can be concluded that the policy is detrimental to the environment [23].

As a supranational institution, the EU promotes its member nations' economic and social development. The EU is committed to a greener future. The EU's 2020 environmental plan is a collective national government agenda. Additionally, the EU aims to be more sustainable by 2050 [24]. EU member states must implement National Energy and Climate Plans under the Climate and Energy Act. The EU's 10-year National Energy and Climate Plans (NECPs) aim to meet its energy and climate goals by 2030. Romania and other developing EU members have less national capacity to attain goals than advanced countries.

Romania faces significant challenges and issues related to waste management and pollution, resulting in its ranking as the 25th most problematic country in both areas [25]. Romania's objective is to achieve a 40 percent reduction in emissions by the year 2030 [26]. Considering Romania's status as a developing nation, this figure is remarkably feasible. Romania has adopted the ETS policy due to its membership in the European Union.

European Commission assessment of Romania's National Energy and Climate Plan in April 2020. Romania must reduce 2019 CO2e emissions by 113 Mt. Romania is sponsoring a €1.5 billion indirect emissions reimbursement plan for solid. Laws to prevent carbon leakage in energy-intensive enterprises prompted this strategy. The EUsupports the idea because it fulfils ETSState Aid Guidelines. Romania cut emissions significantly. In 2005 and 2019, Romania cut emissions 26 % faster than the EU average [27]. Romania also contributed 3 % to EU greenhouse gas emissions. Romania implemented land-use and forestry measures in 2018, reducing 24 MtCO2e. Romania achieved good economic growth, reduced emissions, and enhanced well-being in 2021–2022. However, economic growth fell 1.7 percent in the first half of 2023. Romania's medium-term economic growth is expected to be strong [28]. Indeed, Romania is classified as a developing nation that faces technological limitations. However, Romania has benefitted from assistance and promotion from the supranational bodies of the European Union, of which Romania is now a member. By providing financial resources and technological support, Romania will be able to enhance its trading capabilities and foster cooperation. In contrast to China, a relatively recent entrant, it must adjust its utilization and develop novel technology.

China and Romania have quite different ETS systems. China's ETS first targeted the 26,000-ton coal and gas power industry. It then expanded to more industries. These include aviation, process, and power plants. Additionally, China's ETS regulatory system focuses on achieving government-automated domestic policies. Unlike Romania, the EU develops legislation and the European Commission and member states supervise them. As its largest export market, China has a more mature market. The success of seven provinces in implementing the national ETS serves as a measure of China's boldness. The political jurisdiction in China begins at the local level to ensure the smooth execution of policies at the national level [29]. Below is a brief overview of the pilot implementation of the Emissions Trading System (ETS) in seven provinces.

1. ETS implemented in Hubei

Environmental benefits have resulted from the Hubei pilot ETS's 324 million tons of CO2 reduction. Hubei has actively promoted numerous benefits. Companies under its power will follow the rules and stabilise carbon prices by making such a reservation. The Hubei government covers 8 % of the total gain. However, the company cannot spend more than 30 % on carbon price stability. In Q1 2014, Hubei carbon market emissions trading began. By the end of March 2015, the almost one-year-old China Hubei Emission Exchange (CHEX) had stopped trading. The firm traded 8.36 million metric tons of CO2. It represents 43 % of the trading volume of the seven carbon market prototypes.

2. ETS implemented in Guangdong

Starting in 2013, the ETS was extended to electricity, cement, iron, steel, and petrochemical firms. They accounted for 54 % of Guangdong’s CO2 emissions in 2013. Guangdong's law uses the industry's average emission intensity as a benchmark and adds annual emission reduction factors to the allocation process. It limited allowances and incentivized those who completed obligations to reduce emissions. The Guangdong coal market began emissions trading in late 2013. In July 2015, 20.58 million metric tons of CO2 were traded. The seven pilot ETS programs had 36.12 % and 45.28 % transactions worth 918 million yuan.

3. ETS implemented in Beijing

In Beijing, there are two types of enterprises involved in ETS operations: the reporting company and the protected company. Companies with yearly energy use over the specified threshold of 2000 tons are required to disclose their emissions to the government. Protected companies are defined as companies that are significant polluters, with yearly carbon dioxide (CO2) emissions of 10,000 tons or greater. Protected companies are required to manage and regulate their carbon dioxide emissions in compliance with the restrictions set by the Beijing Emissions Trading System (ETS).

4. ETS implemented in Tianjin

Tianjin initiated the implementation of emissions trading in December 2013. Emission allowance trading can be conducted via contracts, spot auctions, or online auctions facilitated by the Tianjin Climate Exchange (TCE). The CO2 quota in Tianjin has undergone significant price volatility following the commencement of the coal market. In March 2014, the cost of The Tianjin coal quota reached its peak at 50.11 yuan per ton of carbon dioxide (tCO2), whereas in June 2017, the Tianjin ETS recorded its lowest value at 8 yuan per tCO2. The price volatility should not surpass 10 %.

5. ETS implemented in Shenzhen

Technology-based firms and high-tech services dominate Shenzhen's building, transportation, and industry sectors, which emit CO2. Shenzhen ETS includes 635 factories and 194 public buildings. Shenzhen's 2010 CO2 emissions were 31.73 million metric tons of CO2 equivalent (MtCO2e), 38 % of its total. Shenzhen ETS allows CCER (China Certified Emission Reduction) to offset up to 10 % of emissions. Individuals who violate the regulations will be fined three times the average market price for excess emissions. In the next year, their quotas will be cut, and their financial support will end.

6. ETS implemented in Shanghai

The Emissions Trading System (ETS) in Shanghai employs the benchmark approach and designates a portion of the benefits to be distributed free of charge. However, the government also proposes that a portion of the benefits be auctioned off to assist the company in meeting its obligations. Shanghai-based companies are restricted to utilizing only 5 % of the entire emission allowance of CCER (Carbon Credit Emission Reduction).

7. ETS implemented in Chongqing

Chongqing is a metropolis with a strong industrial base, relying on manufacturing to drive its economy. Manufacturing enterprises in this city have accounted for 70 % of Chongqing city's Gross Regional Product (GRK). The feature implemented at the Chongqing ETS aim to restrict the number of annual allowances, preventing protected firms from selling emission allowances without any productive outcome. The maximum threshold is 50 % of the yearly complimentary allowance.

According to the aforementioned facts, the implementation of ETS can be feasible even in nations that have a single-party system (communist). An intriguing phenomenon arises from Romania's historical position as a communist state within the European Union, which operates under a supranational structure. However, it is widely recognized that communist states tend to be hesitant to embrace standards that emanate from Western nations, particularly those with liberal ideologies. However, China and Romania is highly committed to addressing the economic environment, which is the prevailing global standard of governance. We firmly believe that global governance can be implemented within a cross-political ideology.

### 6.2 The China environmental economic development challenges

The lack of efficiency in market operations has a detrimental influence on the effectiveness of the Emissions Trading System (ETS). Hence, ETS urges enterprises to persist in their innovation efforts by augmenting both product and operational expenses. Undoubtedly, this nation of pandas possesses the biggest carbon emissions on a global scale. It demonstrates China's capacity to establish a clean growth mechanism. In 2011, China introduced the ETS as a means to incentivize the reduction of their carbon emissions. As of 2013, the ETS pilot project has been initiated in nine provinces and cities. Subsequently, in June 2021, the national ETS was formally launched (W. [2]).

ETS openly acknowledges that its investment regulatory framework is changing due to increased carbon emission mitigation costs. Regulated enterprises must invest more to reduce carbon emissions and comply with manufacturing requirements. It complicates and affects the company's growth strategy. In fact, company investment drives economic growth. State-owned firms, which understand China's politics, are unaffected by such scenarios. These environmental criteria greatly impact business competitiveness and investment patterns. Thus, governments must actively promote carbon markets to reduce policy costs.

Undoubtedly, this is an unfavourable indication of China's economic expansion as it carries enduring consequences that produce outcomes. Hao Ran Zhang demonstrated the efficacy of ETS in achieving a harmonious balance between carbon reduction and economic development objectives using the differential method (DID) and the synthetic control method (SCM). In locations such as Hubei, Tianjin, and Chongqing, the emission intensity is down while the GDP is increasing. ETSs pertaining to economic growth are not incompatible but rather complimentary. Implementing an ETS policy can enhance energy efficiency and facilitate the transfer of resources from industry, leading to significant improvements in energy efficiency and overall sustainability. Furthermore, these policies can foster and augment the capacity of states or corporations to generate low-carbon progress [15].

ETS, company innovation, and financial performance are believed to be interconnected, as suggested by specific experts. At this microscopic level, the improvement in financial performance is uncertain. Hence, in addition to focusing on emission reduction, corporations need to take into account their economic performance. The implementation of appropriate policies can amplify the economic advantages of environmental investment in China's ETS. Investments with a low-carbon focus could potentially enhance the returns on shares. ETS can enhance the company's overall profitability and mitigate capital expenses. Reducing carbon emissions can enhance companies' performance and yield economic benefits.

Ownership rights are possessed by all companies. These disparities impact the extent of social accountability, attributes, and worth of the management's inclination towards economic endeavours. Private corporations and state-owned enterprises each have their own distinct regulations. Companies must exhibit distinctiveness in their cognitive processes and behaviors. Therefore, the government must function as a regulatory entity by actively pushing the implementation of policies. The enforcement of ETS is intricately connected to the ownership rights of either a state-owned enterprise or a privately owned corporation. The variation in ownership results in varying degrees of implementation of ETS.

Due to China's insufficient decentralization of government and enterprises, Wanyi Chen noted that SOE is heavily regulated. Thus, SOEs must connect their values with the governments to achieve a common goal. SOEs must participate in the market to implement the ETS. The company's operations must be upgraded to reduce carbon emissions. The company is more socially responsible than private firms. Private companies lack the incentive to change their investment structures and trade carbon when given market-based environmental regulatory instruments (W. [2]). Private and government-owned enterprises have different governance and goals. A poorly developed ETS strategy affects environmental regulations.

Another differentiating factor is the varying levels of investment efficiency that can be attained by state-owned enterprises and privately held businesses. Due to receiving governmental assistance and securing superior finance, SOE enjoys significant advantages. The government is responsible for running, supervising, and organizing all operations (W. [2]). State-owned enterprises have the ability to promptly modify their investment framework following the implementation of the ETS. In order to enhance the efficacy of SOE investments, they modified their original investment framework to incorporate carbon trading. Compared to more stringent private company finance, which poses challenges for involvement in the ETS, Wanyi Chen argues that the adoption of ETS has a limited impact on the effectiveness of private corporate investment in comparison to SOE.

The implementation of ETS also poses challenges for minimal investment. ETS recommends that companies utilize technology and prioritize investments in efficiency rather than focusing solely on size. The externality of ETS mitigates the agency problems faced by the corporation. Wanyi Chen's quantitative differences-in-differences (DID) analysis has shown that overinvestment has a negative impact on state-owned enterprises (SOEs), despite their overall performance. Private corporations, being affluent, do not engage in excessive investment. Cost effects typically lead to higher investment and lower output. Businesses have traditionally acquired carbon allowances through economic means. This is distinct from enhancing corporate research and development efforts in order to reduce carbon emissions. Wanyi Chen's analysis included a table that displayed a Treat∗Post coefficient of −0.019 to describe the data. These statistics suggest excessive investment in state-owned enterprises. The effectiveness of investment is significantly influenced by environmental, social, and governance requirements.

Examining the implementation of the ETS in China reveals challenges about business institutions and time periods. Within the realm of business institutions, the roles assumed can be categorized as either private or state-owned enterprises. Both of these parties possess distinct requirements and challenges when it comes to executing this ETS. In regards to the timeframe, the ETS has a detrimental effect in the immediate period but provides advantages in the distant future. Both corporate institutions and the timeframe influences the implementation efficiency of this ETS. These two prerequisites appear contradictory since the government is required to adhere to the Sustainable Development Goals (SDGs) while still pursuing economic advancement.

Subsequently, the following chapter delves into the topic of political authority. Implementing global norms in a communist-based society is easier because the norms align with the country's issues and interests. In theory, the political authority employs a strategy to ensure compliance with all policies established by the central government.

### 6.3 Analyzing China’s ETS implementation with a political authority approach and neo-marxist perspective

Given the presence of these challenges, it is only natural to anticipate a favourable conclusion for the Chinese country. The correlation between Chinese political authority and the attainment of China's environmental and economic goals is related. For instance, a province in China is solely interested in augmenting local revenue without taking into account the long-term viability of connections. Undoubtedly, this will disrupt the ongoing implementation of the existing policy. China's political authority is sufficiently robust to effectively compel the actors and institutions involved to adhere to the ETS policy. Given the Chinese state's stringent regulations and established standards, the implementation of any policy will be readily accomplished. ETS offers prospects for company investment through innovative models and fosters fresh competition in the business sector. The ensuing competition will yield products of high quality. Nations would actively engage in competition to augment their revenue by implementing regulations such as ETS or even adopting ETS as a policy measure.

The examination primarily focuses on the Chinese government's fiscal policy and its connection to development strategies that are centered around environmental economics and political authority. There are two kinds of CO2 reduction initiatives that can be undertaken: government-led and market-driven. Within a market-oriented framework, the techniques that can be employed include carbon trading and carbon taxes, whereas government-driven solutions entail the allocation of fiscal resources towards the development of a low-carbon economy. In addition, there are ongoing efforts to enhance energy efficiency and optimize energy structures, which undoubtedly incur substantial expenses [30]. The effect of fiscal spending on lowering carbon emissions is yet distant. However, it is crucial to consider the entirety of the subject matter across multiple dimensions and timeframes rather than solely focusing on immediate circumstances. Fiscal expenditure measures exhibit a detrimental impact in the short term and a beneficial effect in the long term[31].

China's commitment to reducing carbon emissions is seen in its financial assistance for critical projects, tax incentives, industrial initiatives, and investment strategies. China employs taxation as a means to incentivize desirable behaviors such as environmental conservation and prudent resource utilization while simultaneously discouraging undesirable outcomes such as pollution and carbon emissions. Carbon pricing incentivizes enterprises to use more environmentally friendly production technologies and processes in order to reduce their carbon emissions. The transition to a low-carbon economy frequently results in a decrease in air pollution due to the interconnected nature of many emission sources [32]. China can to impose taxes on several aspects, such as environmental preservation, resource utilization, specific vehicle and ship purchases, and general product consumption. China possesses law enforcement and punitive procedures. China, as a communist state, has the authority to compel corporate entities to adhere to its regulations under the current policies. Most Chinese carbon designs provide that the company will face fines ranging from one to three times the market price in the event of excessive emissions.

According to environmental economics theory and law enforcement mechanisms, the ETS can be considered the most efficient environmental regulating tool, capable of fostering a well-balanced economy. It is essential to acknowledge that the deployment of ETS can have adverse effects on the company's short-term outcomes. This influence arises from the company's endeavour to modify its production patterns, resulting in the company encountering difficulties and challenges. Their operational activities decelerated, resulting in a subsequent deceleration in economic growth in China (Xiaohui Yang et al., 2021). The crucial aspect is the ability of an ETS to endure and evolve into a sustainable policy, thereby exerting a significant influence in the future.

The utilization of political authority to enforce policies might expedite the adjustment of the individuals concerned towards the effective implementation of these policies. Consequently, China possesses a formidable political authority to compel its citizens and organizations to adhere to the regulations of the ETS. China's robust social control exemplifies its adoption of a state-centric approach towards the establishment and implementation of laws and policies. Within the framework of social control, business institutions are obligated to adhere to established regulations while engaging in their commercial endeavours. Violating the rules incurs penalties. China not only regulates institutions but also empowers its citizens to exercise social control by promoting domestic and ecologically friendly products. Achievement of public consciousness about the utilization of eco-friendly items was ultimately established.

Economic growth and a healthy ecosystem emerge from this success. If a policy fails to benefit all parties, it is likely harmful to the environment. The communist state has its own stringent laws and regulations pertaining to budgetary policy. Fiscal policy is primarily directed towards facilitating a positive shift in the country's economic structure. The communist regime has implemented several measures, including the privatization of state-owned firms. Additionally, it has the potential to generate cost savings by advocating for a decrease in defense expenditures and shifting away from prioritizing geopolitical spending. Tax and budget changes have also emerged as crucial macroeconomic measures for the nation. By implementing fiscal policy measures, the communist state enhances economic vitality and stimulates investment activity, even in the face of fluctuations [33].

China currently demonstrates an enhanced production efficiency of its companies, both in the short term and in the long term. The resulting products become more competitive, hence contributing to an improvement in China's economic level. Not only democratic countries but also communist ones, like China, may effectively implement environmental policies by coercive means for the sake of environmental interests. Business actors must adhere to China regulations and policies in accordance with the desired system. China's environmentally conscious business and innovation strategy is experiencing significant growth, reaching 90 %. This is a result of heightened consumer consciousness regarding the environment. China will experience significant economic growth as a result of the creation of a vast market.

China has successfully implemented local political authority in all regions. Based on adherence and industry, each region's coercive policy is different. ETS implementation can be similar to EU standards by implementing enforceable regulations and substantial penalties. Neo-Marxist thinkers believe the state formulates and implements policies independently. This scenario executes all actions state-centrically. Neo-Marxism and political authority are linked. Being a communist nation helps China execute its policies and achieve its goals. China's state-centric political authority can maximize the ETS. With its economic and political authority, the Communist Party of China (CCP) is important to the government. Party leaders set national policies, implement programs, and oversee varied societies [13].

The leaders implemented a program in multiple regions in China that ultimately demonstrated success in positively impacting the economy and reducing carbon emissions. Companies operating in some regions of China are obligated to adhere to the established regulations and legislation. The implementation of the ETS has been expedited by China's state-centric strategy, disregarding regional regulatory disparities. The Chinese government is highly resistant to external interference, notwithstanding the vulnerability of Chinese corporations to interference. Private enterprises might look to SOE China as a model for strengthening their adherence to policy. Furthermore, as previously mentioned, SOEs are inherently more lucrative due to their enhanced ability to conform to any laws implemented by China.

## 7 Conclusion

This research has addressed the problem statement by identifying the following variables: a) ETS China aligns with the principles of environmental economics, and b) ETS China is well-structured. The findings suggest a positive correlation between China’s economic growth and carbon emissions reduction, leading to a mutually advantageous outcome. It can be demonstrated that investment activities in China are increasing, and there is technology innovation, as well as a decrease in the overall annual carbon emissions. In addition, the ETS policy is implemented systematically by designating its seven provinces as ETS pilots. Through this achievement and learning from the past, China was able to successfully implement a nationwide ETS with favourable outcomes. China successfully incorporates existing regulations by adapting to established rules and guidelines.

This article analyses China's industry sector from the perspective of environmental economics, as indicated by the statistics above, which reveals that Treat∗Post SOE recorded a value of −0.019. This signifies that SOE has attained the highest investment rate by implementing the ETS. Conversely, the private sector incurs slightly higher expenses in adhering to the ETS regulation, although this ultimately boosts investment activity in the private sector. China is reliant on international markets to promote and sell its industrial products. China not only focuses on promoting product but also prioritizes compliance with environmental regulations, particularly those related to emissions. In order to do this, China needs stringent emissions laws that both state-owned enterprises and the private sector can implement.

China, as a communist nation, continues to uphold its state-centered approach, characterized by solid political authority in implementing policies within the framework of ETS. The practice of global governance greatly relies on the enforcement of robust laws and regulations for the sake of national interests and long-term effects. Implementing the ETS may not always have a negative impact on the economy, but it can actually be beneficial when the country has a robust regulatory framework. The economic growth and reduction in emissions are co-occurring through implementing a state-centric ETSpolicy supported by China's strong political authority.

The future challenge for China lies in how effectively it can balance economic expansion and environmental objectives. This research will likely lead to further studies that can develop a model capable of integrating environmental sustainability with economic growth. There are still many concepts and approaches that can be utilized by future research to strengthen the argumentation on this issue.

## CRediT authorship contribution statement

**Roseno Aji Affandi:** Writing – review & editing, Writing – original draft, Visualization, Validation, Supervision, Project administration, Conceptualization. **Adrian:** Writing – review & editing, Writing – original draft, Resources, Methodology, Investigation, Formal analysis, Writing – review & editing, Writing – original draft, Resources, Methodology, Investigation, Formal analysis.

## Declaration of competing interest

The authors declare that they have no known competing financial interests or personal relationships that could have appeared to influence the work reported in this paper.
